# Mussel-Inspired Multiwalled Carbon Nanotube Nanocomposite for Methyl Orange Removal: Adsorption and Regeneration Behaviors

**DOI:** 10.3390/molecules29153535

**Published:** 2024-07-27

**Authors:** Yongjian Jiang, Erqiang Sun, Fengyang Zhao

**Affiliations:** 1 College of Sciences, Liaoning Petrochemical University, Fushun 113001, China; 2 School of Petrochemical Engineering, Liaoning Petrochemical University, Fushun 113001, China; q1559667016@163.com

**Keywords:** mussel-inspired chemistry, multiwalled carbon nanotubes, catechol, polyethyleneimine, adsorption

## Abstract

A mussel-inspired multiwalled carbon nanotube (MWCNT) nanocomposite (MWCNTs@CCh-PEI) was prepared by the co-deposition of catechol (CCh)/polyethyleneimine (PEI) and modification of MWCNTs for the efficient removal of methyl orange (MO). The effects of MO solution pH, contact time, initial MO concentration, and temperature on the adsorption capacity of MWCNTs@CCh-PEI were investigated. The results indicate that the adsorption capacity of MWCNTs@CCh-PEI was two times higher than that of pristine MWCNTs under the same conditions. The adsorption kinetics followed the pseudo-second-order model, suggesting that the adsorption process was chemisorption. The adsorption isotherm shows that the experimental data were fitted well with the Langmuir isotherm model, with a correlation coefficient of 0.9873, indicating that the adsorption process was monolayer adsorption. The theoretical maximum adsorption capacity was determined to be 400.00 mg·g^−1^. The adsorption thermodynamic data show that the adsorption process was exothermic and spontaneous. More importantly, the adsorption capacity of MWCNTs@CCh-PEI showed no significant decrease after eight reuse cycles. These results demonstrate that MWCNTs@CCh-PEI is expected to be an economical and efficient adsorbent for MO removal.

## 1. Introduction

With the development of the economy and rising demand for textile products, a large amount of dye wastewater is produced every year worldwide. The dye effluents with high chroma, complicated composition, non-biodegradability, and high chemical oxygen demand cause severe damage to the ecological environment. Many azo dyes have high biological toxicity, which risks carcinogenesis, teratogenesis, and renal dysfunction [1,2]. At present, various technologies, such as solvent extraction, membrane separation, electrochemical precipitation, biological treatment, and adsorption, have been reported to remove organic dye pollutants in water systems [3]. Among them, adsorption has unique application potential in dye wastewater treatment due to its advantages of simple operation, economy, and high efficiency [4]. For the adsorption process, it is crucial to select the appropriate adsorbent to remove dye pollutants. So far, various adsorbents for dye contaminants removal, such as activated carbon [5], silica [6], Mxene [7], polymers [8], and MOFs [9], have been reported. Unfortunately, the industrial application of most adsorbents to cope with the increasing volume of dye pollutants is very challenging due to their insufficient adsorption capacity, which is time-consuming and non-renewable. Therefore, it is urgent to develop a large-scale sustainable adsorbent that can remove dyes rapidly and efficiently.

Multiwalled carbon nanotubes (MWCNTs) are one of the most popular nanomaterials made from carbon. MWCNTs have been widely used in dye adsorption owing to their unique hollow structure, excellent mechanical properties, and high specific surface area [10,11]. However, there are strong hydrophobic interactions between pristine MWCNTs, which tend to aggregate in water, resulting in significantly reduced specific surface areas. Meanwhile, the surface of pristine MWCNTs lacks active functional groups, which seriously affects the further application of MWCNTs in the adsorption field. Thus, it is necessary to adopt physical or chemical methods to introduce the active functional groups (such as NH_2_, -OH, and -COOH) on the surface of pristine MWCNTs to improve their adsorption capacity for dyes and enhance their application in environment treatments [12,13,14].

In recent years, mussel-inspired chemistry has been widely applied to the surface functionalization modification of adsorbents because of its advantages of simplicity of operation, high efficiency, eco-friendliness, and mild reaction conditions [15,16,17]. Its reaction mechanism is that natural phenolic compounds, such as the most representative dopamine (DA), form polyphenolic aggregation spontaneously under aerobic and alkaline conditions. These aggregates can be deposited on various substrates to form adhesive coating layers, accomplishing the surface functionalization of nano-adsorption materials. For example, Zhang et al. [18] synthesized polydopamine (PDA)-modified MWCNTs (P-MWCNTs), which exhibited better methylene blue (MB) adsorption capacity in alkaline environments compared to MWCNTs. Wang et al. [19] reported a new magnetic nano-adsorbent for smartly selective dye removal via the simultaneous combining of PDA and polyethyleneimine (PEI) on Fe_3_O_4_ nanoparticles. However, the high cost of DA limits its large-scale application in the adsorption field, and finding cheaper phenolic compounds to modify nano-adsorption materials will be the primary task for industrial production [20,21].

Compared to DA, which is expensive and requires strict storage conditions, catechol (CCh) is a cost-effective and storage-friendly phenolic compound capable of oxidative self-polymerization under aerobic and alkaline conditions. Its polymerization process is also conveniently regulated [3]. However, the lack of amino groups in CCh compared to DA results in a weaker interaction between the polyphenol and the modified substrate [22]. Nevertheless, the abovementioned shortcomings can be effectively alleviated by incorporating polyamine compounds into the reaction system [23]. Therefore, replacing DA with CCh and polyamine compounds is very suitable for the large-scale preparation of nano-adsorption materials by mussel-inspired chemistry.

In this study, a binary system of low-cost CCh and polyethyleneimine (PEI) was used to replace DA to modify pristine MWCNTs by a one-step co-deposition method. A novel MWCNTs nanocomposite (MWCNTs@CCh-PEI) with excellent adsorption properties was successfully prepared. The pristine MWCNTs and MWCNTs@CCh-PEI were characterized by FTIR, TEM, SEM, TGA, and BET. We selected the most typical azo dye, methyl orange (MO), as the target contaminant. The adsorption performance of MWCNTs@CCh-PEI was investigated using different MO solution pH, contact time, initial MO concentration, and adsorption temperature. In addition, the reusability performance of MWCNTs@CCh-PEI was systematically studied.

## 2. Results

### 2.1. Characterizations

**Figure 1:**
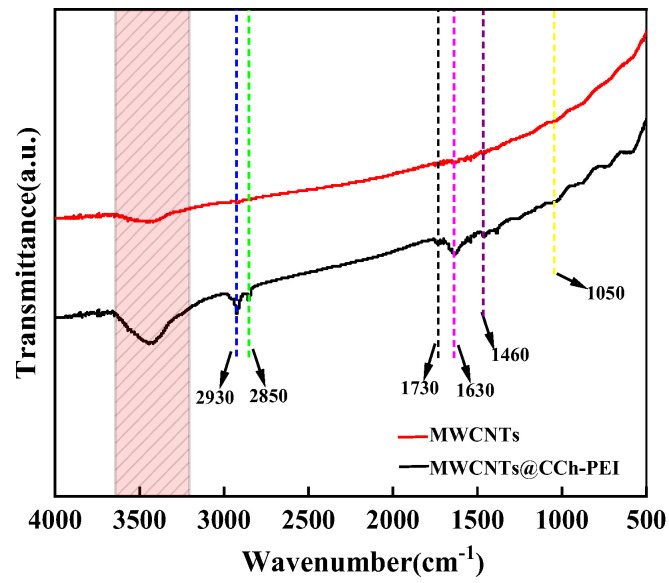
FT-IR spectra of pristine MWCNTs (Red line) and MWCNTs@CCh-PEI (Black line).

The chemical structures of pristine MWCNTs and MWCNTs@CCh-PEI were characterized by FT-IR, as shown in Figure 1. The FT-IR spectrum of MWCNTs@CCh-PEI showed an intense broad absorption band observed at 3600~3200 cm^−1^, attributed to a superposition of N-H and O-H stretching vibrations. Also, 2930 cm^−1^, 2850 cm^−1^, and 1460 cm^−1^ are attributed to the absorption bands of saturated C-H stretching and bending vibrations from PEI molecules, respectively [24]. A new characteristic absorption band at 1730 cm^−1^ corresponds to a C=O stretching vibration in the quinone group [21]. The absorption peak at 1630 cm^−1^ is assigned to the C=N stretching vibration formed by the Schiff base reaction between CCh and PEI [25]. Meanwhile, a new peak at 1050 cm^−1^ may be due to the ether group formed by the self-polymerization of CCh [26]. It can be concluded that the co-deposition of CCh and PEI on the surface of MWCNTs via mussel-inspired chemistry is successful.

**Figure 2:**
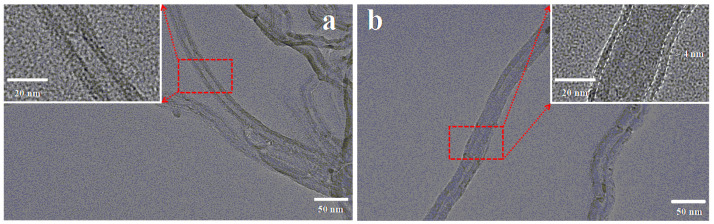
TEM images of pristine MWCNTs (**a**) and MWCNTs@CCh-PEI (**b**).

The surface morphologies of pristine MWCNTs and MWCNTs@CCh-PEI were examined using TEM and SEM. Figure 2 shows the TEM images of pristine MWCNTs and MWCNTs@CCh-PEI. It can be seen that the outer diameter of pristine MWCNTs was approximately 20–30 nm (Figure 2a). Figure 2b reveals that the thin polymer layer coated the outer surfaces of MWCNTs with a thickness of about 4 nm. The tubular structure of pristine MWCNTs was not destroyed, illustrating the successful co-deposition of CCh and PEI on pristine MWCNTs via mussel-inspired chemistry, as reported by other studies [27,28].

**Figure 3:**
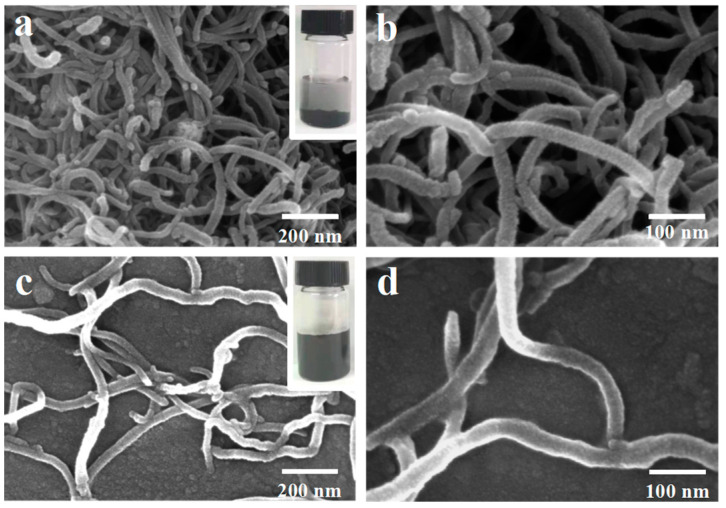
SEM images of pristine MWCNTs (Insert: dispersion of pristine MWCNTs after Scheme 30 min) (**a**-low magnification image, **b**-high magnification image) and MWCNTs@CCh-PEI (Insert: dispersion of MWCNTs@CCh-PEI after standing for 30 min) (**c**-low magnification image, **d**-high magnification image).

Figure 3 is the SEM and dispersion images of pristine MWCNTs and MWCNTs@CCh-PEI. It can be observed that the pristine MWCNTs are tightly entangled with each other due to the massive π-π conjugate interactions between the tubes (Figure 3a,b). As shown in Figure 3c,d, MWCNTs@CCh-PEI has excellent dispersibility compared to the pristine MWCNTs, indicating that the CCh-PEI layer of MWCNTs@CCh-PEI not only weakens the π-π interaction among tubes but also introduces numerous hydrophilic functional groups. The enhancement of dispersibility can make the surface of MWCNTs@CCh-PEI more fully exposed in the dye solution and thus elevate its adsorption capacity for MO.

**Figure 4:**
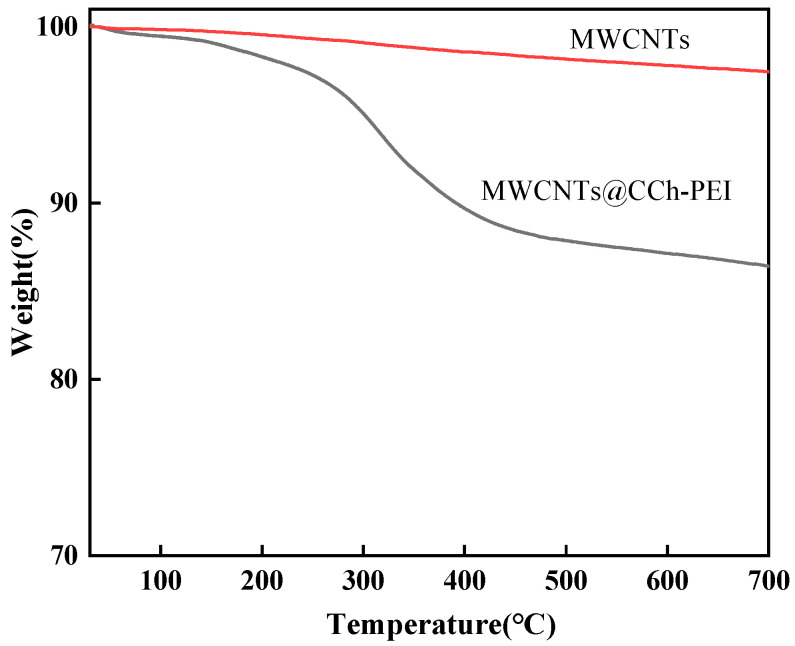
TGA curves of pristine MWCNTs and MWCNTs@CCh-PEI.

TGA was used to estimate the thermal stability of pristine MWCNTs and MWCNTs@CCh-PEI. As shown in Figure 4, it can be observed that pristine MWCNTs did not show a significant mass change trend from 30 to 700 °C. In the same temperature range, MWCNTs@CCh-PEI lost about 13.58% of its weight. The 1.69% weight loss from 30 to 200 °C is due to the volatilization of physically adsorbed water. The apparent 10.44% weight loss from 200 to 500 °C is attributed to the decomposition of polymers formed by CCh and PEI [29]. Moreover, approximately 1.45% of the weight loss is due to the decomposition of amorphous carbon or impurities until the temperature reaches 700 °C. The above analysis indicates that MWCNTs@CCh-PEI was successfully prepared.

**Figure 5:**
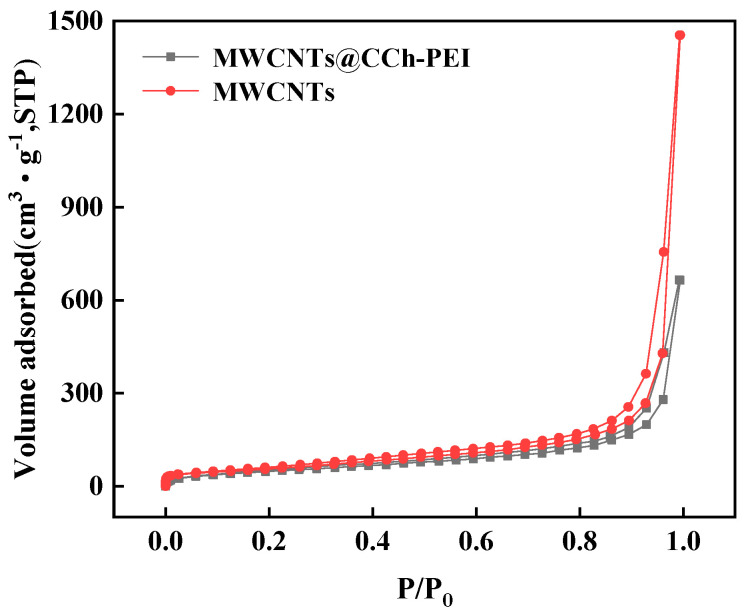
N_2_ adsorption–desorption isotherm of pristine MWCNTs and MWCNTs@CCh-PEI.

N_2_ adsorption–desorption analysis was performed on MWCNTs before and after modification. Figure 5 shows the adsorption and desorption curves of N_2_ for MWCNTs and MWCNTs@CCh-PEI. Table 1 shows the specific surface areas of MWCNTs and MWCNTs@CCh-PEI calculated by the BET method. As can be seen from Figure 5, the N_2_ adsorption–desorption curve of MWCNTs@CCh-PEI was slightly lower than that of MWCNTs, indicating that the specific surface area of MWCNTs was slightly reduced after modification. According to the data in the table, the total pore volume and average pore size after modification decreased from 2.3 cm^3^ g^−1^ and 4.1 nm before modification to 1.0 cm^3^ g^−1^ and 2.2 nm, respectively, indicating that some micropores and mesoporous pores of MWCNTs were occupied by copolymers formed by the reaction of CCh and PEI during the modification process. The total pore volume and average pore diameter of the material were reduced. At the same time, the formation of amino-containing coatings on the surface of MWCNTs reduced the specific surface area of the material. Fortunately, the presence of many amino groups also provided numerous binding sites for the prepared MWCNTs@CCh-PEI [30].

### 2.2. Adsorption Properties of As-Synthesized MWCNTs@CCh-PEI

#### 2.2.1. Effect of MO Solution pH on Adsorption Capacity

**Figure 6:**
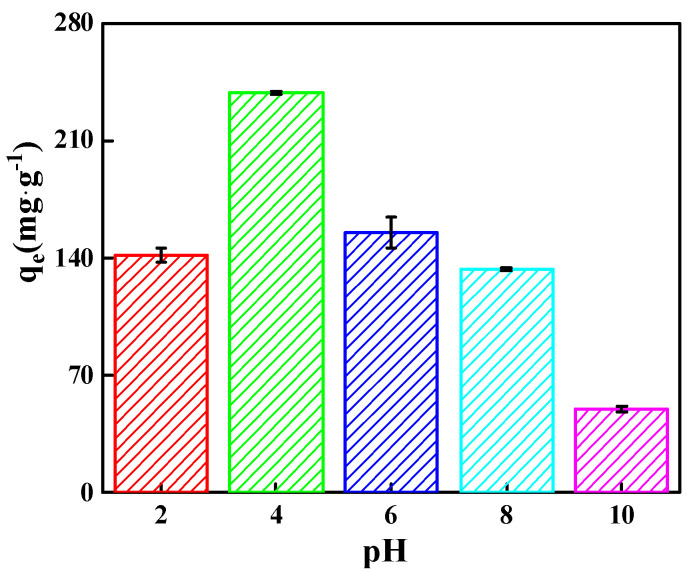
Effect of pH on MO adsorption by MWCNTs@CCh-PEI.

The solution pH impacts the adsorbent’s surface properties/charge density and the dye molecules’ charge/structure. It is a vital element in the dye adsorption process [31]. Figure 6 illustrates the influence of pH on the amount of MO adsorbed on MWCNTs@CCh-PEI. As the solution pH increased from 2 to 4, the absorbed amount of MO increased. This is attributed to the fact that the abundant amino groups on the surface of MWCNTs@CCh-PEI are protonated to positively charged groups (-NH^3+^) at high concentrations of H^+^, which facilitates electrostatic interactions with anionic MO molecules in the aqueous solution [32]. However, the decrease in the amount of adsorbed MO at pH 2 was mainly due to the presence of excess H^+^ ions in the solution, which contributed to the protonation of the sulfonic acid ion (-SO_3_^−^) of MO and the formation of -SO_3_H at a solution pH lower than the pKa (3.4) of MO [3,33], leading to the weaken electrostatic attraction between MO and MWCNTs@CCh-PEI. With the increase in pH of the solution from 4 to 10, MWCNTs@CCh-PEI was gradually deprotonated, decreasing the positive charge density on the surface of MWCNTs@CCh-PEI. The diminished electrostatic interaction between MWCNTs@CCh-PEI and MO leads to lower adsorption capacity. Thus, the pH of the solution was set to 4 in the other experiments [3].

#### 2.2.2. Effect of Contact Time on Adsorption Capacity and Adsorption Kinetics

**Figure 7:**
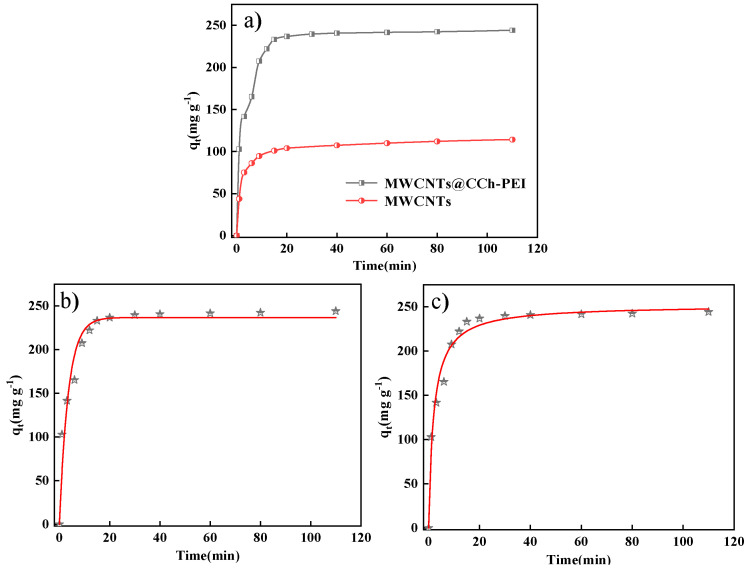
Effect of contact time on MO adsorption by pristine MWCNTs and MWCNTs@CCh-PEI (**a**); pseudo-first-order model (**b**); pseudo-second-order model (**c**).

Figure 7a illustrates the relationship between contact time and the absorbed MO amount on pristine MWCNTs and MWCNTs@CCh-PEI. As shown in Figure 7a, the amount of MO adsorbed onto MWCNTs@CCh-PEI at equilibrium was approximately twice that of the original MWCNTs, and the time required to achieve equilibrium for MWCNTs@CCh-PEI was just 30 min. It may be attributed to the fact that introducing a CCh-PEI coating on the surface of MWCNTs can effectively increase the adsorption capacity and rate of adsorbed MO. In addition, the adsorption process of MO is divided into three main stages: the fast adsorption stage, the slow adsorption stage, and the adsorption equilibrium stage. Initially, there are many adsorption sites on the adsorbent surface, and the concentration of MO is high. The driving force generated by the concentration difference between the adsorbent surface and the solution drove many MO molecules to diffuse rapidly onto the adsorbent surface [23]. As MO molecules gradually occupied the adsorption sites, the driving force generated by the concentration difference diminished, and the adsorption rate gradually decelerated to equilibrium [34]. Hence, follow-up experiments used 60 min as the time required for the MWCNTs@CCh-PEI-adsorbed MO to reach equilibrium.

To further understand the MO adsorption process of MWCNTs@CCh-PEI, the results of fitting the experimental data of adsorption kinetics using pseudo-first-order kinetic (Equation (S1)) and pseudo-second-order kinetic (Equation (S2)) models are shown in Figure 7b,c. The corresponding kinetic parameters and correlation coefficients (R^2^) obtained from the slope and intercept are shown in Table 2. As shown in Figure 7b,c and Table 2, the R^2^ for the pseudo-first-order kinetic model and pseudo-second-order kinetic model were 0.9481 and 0.9788, respectively. By comparing R^2^, it is observed that the R^2^ of the pseudo-second-order was closer to 1, and the calculated equilibrium adsorption capacity q_e_ (cal) was 252.00 mg·g^−1^, which deviated less from the experimental value q_e_ (exp) (238.53 mg g^−1^). The above results suggest that the adsorption process of MWCNTs@CCh-PEI on MO fits well with the pseudo-second-order kinetic model. It indicates that the adsorption is predominantly chemisorption, thereby confirming the robust electrostatic interactions in the MO adsorption process of MWCNTs@CCh-PEI [35,36].

#### 2.2.3. Effect of MO Initial Concentration on Adsorption Capacity and Adsorption Isotherm

**Figure 8:**
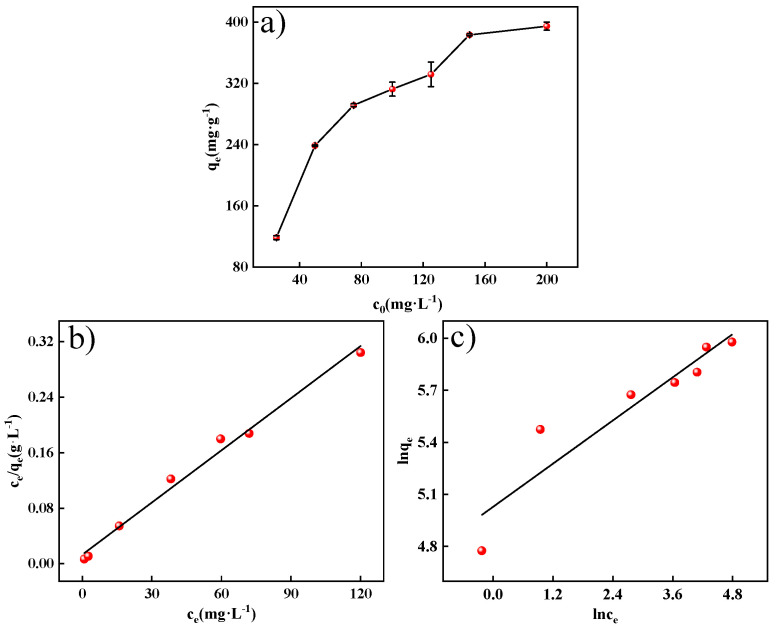
Effect of initial concentration on MO adsorption by MWCNTs@CCh-PEI (**a**); the fitting curve of Langmuir isotherm model (**b**); the fitting curve of Freundlich model (**c**).

The correlation between the initial MO concentration and the adsorption performance of MWCNTs@CCh-PEI is plotted in Figure 8a. With the increase in the initial MO concentration, the amount of MO adsorbed on MWCNTs@CCh-PEI gradually increased and finally became saturated. The adsorption amount reached 394.66 mg·g^−1^ at the initial concentration of 200 mg·L^−1^. The primary reason is that the amount of adsorbed MO is associated with the number of MO molecules migrating to the MWCNTs@CCh-PEI surface. At low initial concentrations, the number of adsorption sites on the MWCNTs@CCh-PEI surface is higher than the number of MO molecules in the solution. Moreover, the concentration difference between the MWCNTs@CCh-PEI surface and the solution is slight, generating driving forces that are not sufficient to fully utilize a large number of adsorption sites on the adsorbent surface. With the initial MO concentration increase, the concentration gradient’s driving force increased, causing more MO molecules to diffuse to the surface of MWCNTs@CCh-PEI. When MO fully occupied the adsorption site on the adsorbent surface, the adsorption quantity achieved saturation and ceased growing. This is consistent with the results reported in the literature [37].

The adsorption isotherms of adsorbed MO on MWCNTs@CCh-PEI were investigated by the Langmuir isotherm model (Equation (S3)) and the Freundlich isotherm model (Equation (S4)). The isotherm plots and fitted parameters are shown in Figure 8b,c and Table 3, respectively. It can be observed that the linearity of the Langmuir adsorption isotherm of MWCNTs@CCh-PEI-adsorbed MO was significantly higher than that of the Freundlich adsorption isotherm. Moreover, the Langmuir model had a higher R^2^ (0.9873) than the Freundlich model (0.8593). Therefore, the Langmuir isotherm model could explain the MO adsorption process of MWCNTs@CCh-PEI. It indicates that the active sites on the surface of MWCNTs@CCh-PEI are uniformly distributed. Meanwhile, the adsorption of MO on MWCNTs@CCh-PEI is monolayer adsorption. The maximum monolayer adsorption capacity obtained from the Langmuir isotherm model was calculated to be 400.00 mg g^−1^ (303 K) and superior to many results in the literature (Table 4). Furthermore, the R_L_ values calculated by Equation (S5) were in the range of 0.0257–0.1742 (0 < R_L_ < 1), indicating the excellent adsorption performance of MWCNTs@CCh-PEI. For the Freundlich model, n was 4.82 (0 < n^−1^ < 1), which indicates that the MO adsorption does not require external energy consumption and is favorable [38].

#### 2.2.4. Effect of Temperature on Adsorption Capacity and Adsorption Thermodynamic

**Figure 9:**
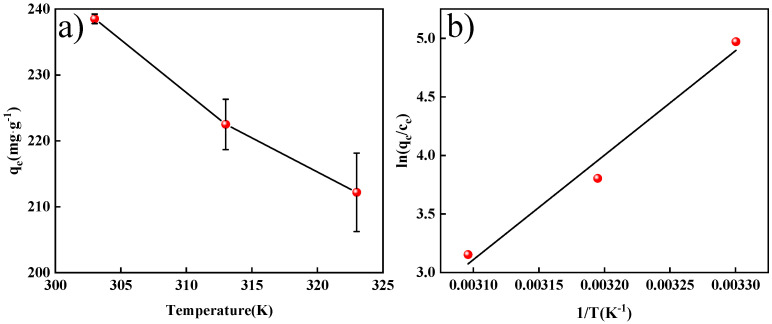
Effect of temperature on the amount of adsorbed MO on MWCNTs@CCh-PEI (**a**); Van ‘t Hoff plots of MO adsorption (**b**).

Temperature can significantly affect the diffusion rate of an adsorbent between solid and liquid phases and is an essential factor in the adsorption process. The effect of temperature on the amount of MO adsorbed on MWCNTs@CCh-PEI is shown in Figure 9. Figure 9a shows that the adsorption capability of MWCNTs@CCh-PEI toward MO decreased from 238.53 to 212.20 mg·g^−1^ as the temperature increased from 303 K to 323 K. The result reveals that increasing the temperature is not favorable for the adsorption of MO by MWCNTs@CCh-PEI. Two main reasons can be used to explain this phenomenon. On the one hand, increasing the temperature will accelerate the diffusion rate of MO molecules from the adsorbent surface into the solution. On the other hand, high temperatures increase MO solubility, promoting the interaction between MO molecules and the solvent [47,48].

The thermodynamic parameters of MO adsorption on MWCNTs@CCh-PEI were calculated using the Van ‘t Hoff curves (Figure 9b) (Equations (S6)–(S8)) and are listed in Table 5. The values of ΔG were negative at different temperatures, indicating that the MO adsorption process by MWCNTs@CCh-PEI proceeds spontaneously. The ΔG increased from −12.34 to −8.26 kJ·mol^−1^ as the temperature increased from 303 K to 323 K, indicating that higher temperatures reduced the spontaneity of MO adsorption. Meanwhile, the negative value of ∆H revealed that the adsorption process of MWCNTs@CCh-PEI toward MO was an exothermic adsorption process. The value of |∆H| was greater than 40 kJ·mol^−1^ (74.17 kJ mol^−1^), proving that a strong electrostatic attraction formed between the MO molecule and the amino-functional groups on the surface of MWCNTs@CCh-PEI. Moreover, the negative value of ∆S indicates an increase in confusion at the solid–liquid interface during the adsorption process [49]. These results suggest that MWCNTs@CCh-PEI is a high-efficiency adsorbent for the removal of anionic dyes.

### 2.3. Regeneration

**Figure 10:**
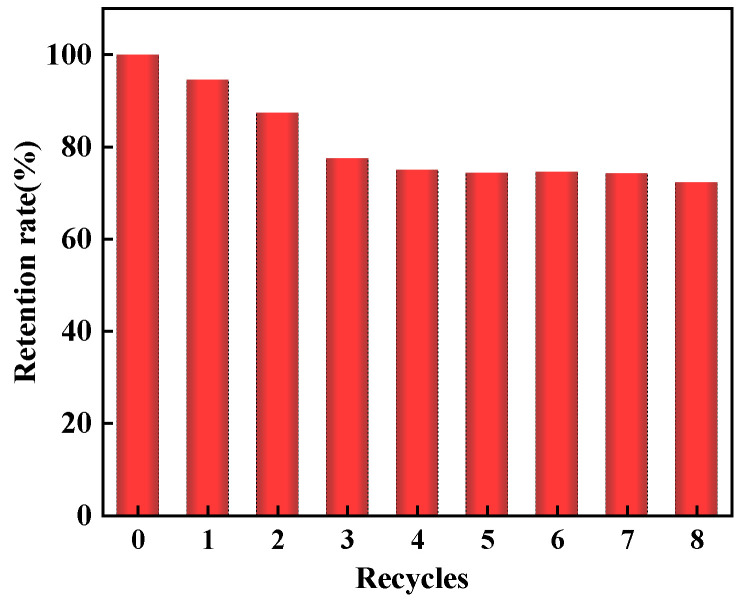
Reusability studies of MWCNTs@CCh-PEI.

The reusability of an adsorbent is a crucial factor in evaluating its practical application. The relationship between the number of adsorption–desorption cycles and the retention rate (RR) of the amount of MO adsorbed on MWCNTs@CCh-PEI is shown in Figure 10. With the increase in the number of cycles, there is a slight decrease in the RR. After eight adsorption–desorption cycles, the RR still exceeded 70%, attributed to the strong interaction between the amino-functional groups on the surface of MWCNTs@CCh-PEI and MO molecules. The above results indicate that MWCNTs@CCh-PEI has excellent reusability. It is expected to be a cost-effective adsorbent for treating actual dye wastewater.

### 2.4. Adsorption Mechanisms

**Figure 11:**
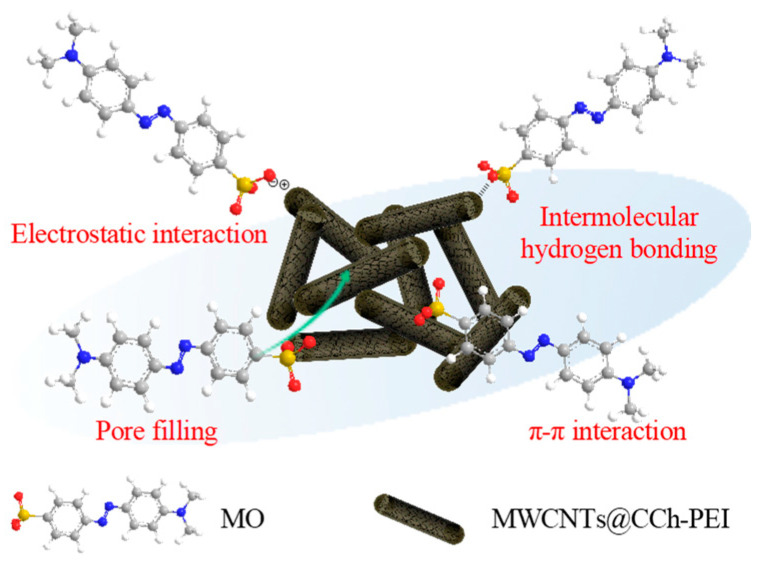
Schematic diagram of the adsorption mechanism.

It is crucial to analyze an adsorbent’s adsorption mechanism to understand its features and adsorption process. The main mechanisms of MWCNTs@CCh-PEI MO adsorption include electrostatic interaction, intermolecular hydrogen bonding, π-π interaction, and pore filling (Figure 11). (1) The amino group on the MWCNTs@CCh-PEI surface showed a positive charge property after protonation, which could afford the adsorption of MO by electrostatic interaction. The result of the effect of MO solution pH on adsorption capacity can support this. Meanwhile, according to the results of kinetic analysis, it can be seen that chemisorption dominates adsorption, which indicates that it is the primary adsorption mechanism [3]. (2) The presence of numerous amino and hydroxyl groups on the surface of MWCNTs@CCh-PEI rendered them capable of adsorption, with MO acting through intermolecular hydrogen bonding [23]. (3) The aromatic ring structure exists in both MWCNTs@CCh-PEI and MO. Hence, the π-π interaction may also promote MO adsorption by MWCNTs@CCh-PEI [41]. (4) There were also some microporous and mesoporous structures in MWCNTs@CCh-PEI, which could also potentially promote the adsorption of MO through pore filling [50].

## 3. Materials and Methods

### 3.1. Materials

MWCNTs (outer diameter = 20–30 nm, length 3–12 µm, 95 wt%) were provided by Nanjing Xfnano, Nanjing, China. Catechol (CCh, 99.5%) and Tris (hydroxymethyl) aminomethane (>99.9%) were obtained from Shanghai Macklin Biochemical, Shanghai, China. Polyethyleneimine (PEI, Mw = 1800, 99%) and sodium hydroxide (NaOH, ≥98.0%) were purchased from Shanghai Titan Scientific, Shanghai, China. Hydrochloric acid (HCl, 36.0–38.0 wt%) was supplied by Tianjin Fuyu Fine Chemical, Tianjin, China. Methyl orange (MO, 96%) was purchased from Shanghai Aladdin Industrial, Shanghai, China. All reagents used in this study were at least of an analytical grade and were used without further purification. All aqueous solutions were obtained with distilled water.

### 3.2. Preparation of MWCNTs@CCh-PEI

**Figure 12:**
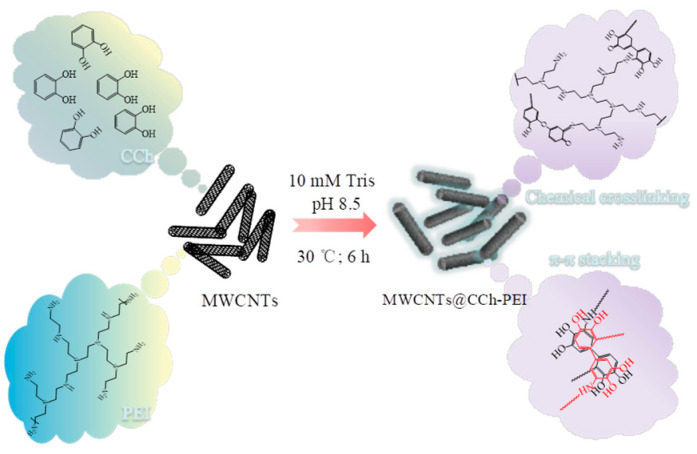
Schematic showing the preparation of MWCNTs@CCh-PEI.

Briefly, 0.5 g pristine MWCNTs were first added into 500 mL of Tris-HCl buffer (10 mmol·L^−1^, pH 8.5) and sonicated at 30 °C for 10 min. Subsequently, 1 g of CCh and 4 g of PEI were simultaneously dissolved to the above dispersion (sonication for 5 min), and the resulting suspension was stirred rapidly at 30 °C for 6 h. Finally, the final black powder product named MWCNTs@CCh-PEI (CCh/PEI mass ratio 1:4) was obtained by filtration of the supernatant (washed with distilled water and alcohol several times) and then dried at 60 °C under vacuum for 12 h (Figure 12).

### 3.3. Characterizations

Morphologies of MWCNTs@CCh-PEI and pristine MWCNTs were visualized by SEM (SU8010, Hitachi, Tokyo, Japan) and TEM (JEM-2000F, JOEL, Tokyo, Japan). Chemical structures were determined with an FT-IR instrument (FT-IR-600+610, Agilent Technologies Inc., Santa Clara, CA, USA) in the range of 4000–400 cm^−1^. Thermal stabilities were characterized by TGA (Q500, TA Instruments, New Castle, DE, USA) within the scope of 30–700 °C (heating rate of 10 °C·min^−1^; nitrogen atmosphere). The N_2_ adsorption–desorption isotherm was determined employing Autosorb-IQ_2_-MP (Quantachrome, Boynton Beach, FL, USA).

### 3.4. Adsorption and Reusability Experiments

The adsorption behavior of MWCNTs@CCh-PEI and the effect of the experimental parameters (e.g., MO solution pH, contact time, initial MO concentrations, and temperatures) on its adsorption capacity were performed in batch adsorption experiments. The effect of CCh/PEI mass ratios was used in 50 mL of MO solution (50 mg·L^−1^, pH 4) with 10 mg of adsorbents at 303 K for 60 min (shaken at 150 rpm). The effect of solution pH was explored by regulating the pH values (2, 4, 6, 8, and 10) of the MO solution using 1.0 mol·L^−1^ of HCl solution or 1 mol·L^−1^ of NaOH. The effect of contact time was studied by detecting the instantaneous MO concentrations at different times (t, min) using a UV-visible spectrophotometer (UV-5500PC, Shanghai Metash Instruments Co., Ltd., Shanghai, China) (0–115 min; λ_max_ = 464 nm). The effect of the initial MO concentrations was investigated by preparing different MO solution concentrations (25–200 mg L^−1^). The effect of temperature was examined at different temperatures (303 K, 313 K, and 323 K) using an air bath thermostatic oscillator. All the experimental data are the averages of triplicate determinations.

The instantaneous and equilibrium adsorption capacity of adsorbents was calculated by the following equations [4]:

(1) $$q_{t}=\frac{\left(c_{0}-c_{t}\right)\cdot V}{m}$$

(2) $$q_{e}=\frac{\left(c_{0}-c_{e}\right)\cdot V}{m}$$

where q_t_ (mg g^−1^) and q_e_ (mg g^−1^) are the adsorption capacity at any time (t) and equilibrium time, and c_0_ (mg·L^−1^), c_e_ (mg·L^−1^), and c_t_ (mg·L^−1^) are the MO concentrations at the initial time, equilibrium time, and any time t (min), respectively. V (mL) is the volume of the MO solution, and m (mg) is the weight of the adsorbent.

The reusability performance of MWCNTs@CCh-PEI was investigated by eight adsorption–desorption cycles. First, 10 mg of MWCNTs@CCh-PEI was mixed with 50 mL of MO solution (50 mg·L^−1^; pH 4) and then shaken at 150 rpm for 120 min (303 K). After adsorption, the collected adsorbent was desorbed by the eluent (2 mol L^−1^ of NaCl solution), subsequently rinsed thoroughly with distilled water, and then dried for 12 h (60 °C). The regenerated MWCNTs@CCh-PEI was reused for the following cycle of MO removal. After each cycle, the retention rate (RR%) of the adsorbent for MO adsorption was calculated according to the following equation:

(3) $$RR=\frac{R_{n}}{R_{1}}$$

where R_1_ and R_n_ are the MO adsorbed onto the adsorbent at the 1st and nth times, respectively.

## 4. Conclusions

Overall, MWCNTs@CCh-PEI was successfully fabricated as a highly efficient nano-adsorbent for MO removal via a mussel-inspired chemistry strategy. The maximum adsorption capacity of 238.53 mg·g^−1^ was obtained, two times higher than that of the pristine MWCNTs under the same experimental conditions (C_MO_ = 50 mg·L^−1^; pH 4; T = 303 K). The results reveal that the adsorption kinetics and adsorption isotherms data obey the pseudo-second-order kinetic and Langmuir isotherm models (Supplementary Materials), respectively. The adsorption process of MO on MWCNTs@CCh-PEI was monolayer chemisorption. The calculated maximum monolayer adsorption capacity was 400.00 mg g^−1^. The thermodynamic data indicate that the adsorption process was exothermal and spontaneous. Additionally, MWCNTs@CCh-PEI exhibited relatively good regeneration behavior, even after eight adsorption–desorption cycles. The above results suggest that MWCNTs@CCh-PEI can potentially be applied to wastewater treatment.

## Figures and Tables

**Table 1:** Specific surface area of pristine MWCNTs and MWCNTs@CCh-PEI.

Samples	S_BET_ (m^2^ g^−1^)	Pore Volume (cm^3^ g^−1^)	Average Pore Diameter (nm)
**MWCNTs**	215.9	2.3	4.1
**MWCNTs@CCh-PEI**	187.1	1.0	2.2

**Table 2:** Pseudo-first-order and pseudo-second-order kinetic model parameters (303 K).

Kinetic Model	Parameters	Initial MO Concentration (50 mg L^−1^)
q_e_ (exp), (mg·g^−1^)		238.53
Pseudo-first-order model	q_e_ (cal), (mg g^−1^)	236.82
	k_1_ (min^−1^)	0.2832
	R^2^	0.9481
Pseudo-second-order model	q_e_ (cal), (mg g^−1^)	252.00
	k_2_ (min^−1^)	0.0020
	R^2^	0.9788

**Table 3:** Adsorption isotherm model parameters of MO on MWCNTs@CCh-PEI.

Adsorption Isothermal Models	Parameters	Temperature (K)
		303 K
Langmuir	K_L_ (L·mg^−1^)	0.19
	q_m_ (mg·g^−1^)	400.00
	R^2^	0.9873
	R_L_	0.0257–0.1742
Freundlich	K_F_ [(mg·g^−1^)(L·mg^−1^)^1/n^]	152.58
	n	4.82
	R^2^	0.8593

**Table 4:** Comparison of adsorption capacity of various adsorbents of MO.

Adsorbents	Maximum Adsorption Capacity (mg g^−1^)	Solution pH	Ref.
Oxygen functionalized CNTs	185.18	---	[39]
Magnesium hydroxide-modified clinoptilolite	99.84	7	[40]
UiO-66	150.60	Neutral conditions	[41]
PNIPAM/magnetite/multiamine-functionalized mesoporous silica composite	225.86	3	[42]
Ni/porous carbon CNTs	271.00	---	[43]
S-doped magnetic mesoporous carbon	114.20	4.5	[44]
Poly(catechol-tetraethylenepentamine-cyanuric chloride)@hydrocellulose	189.39	No regulation	[45]
Mesoporous ZSM-5 zeolite	25.00	1	[46]
MWCNTs@CCh-PEI	400.00	4	This work

**Table 5:** Thermodynamic parameters of MO adsorption on MWCNTs@CCh-PEI.

T (K)	∆G (kJ mol^−1^)	∆H (kJ mol^−1^)	∆S (kJ mol^−1^ K^−1^)
303	−12.34	−74.17	−0.20
313	−10.30		
323	−8.26		

## Data Availability

Data are contained within the article and Supplementary Materials.

## Supplementary Materials

The following supporting information can be downloaded at: https://www.mdpi.com/article/10.3390/molecules29153535/s1, kinetic models, Isotherm models, Thermodynamic models [51,52,53,54,55,56].
