# Supplementary material for: Mussel-Inspired Multiwalled Carbon Nanotube Nanocomposite for Methyl Orange Removal: Adsorption and Regeneration Behaviors
Source: Molecules. 2024 Jul 27;29(15):3535. doi: 10.3390/molecules29153535 (PMC11314330; doi:10.3390/molecules29153535)
Supplement: Supplementary file 1 [file molecules-29-03535-s001.zip › molecules-3108901-supplementary.pdf]

Supplementary Information

Mussel-Inspired Multiwalled Carbon Nanotube Nanocomposite for Methyl orange Removal: Adsorption and Regeneration Behaviors

*Yongjian Jiang[2, *], Erqiang Sun[1], Fengyang Zhao[1, *]*

[1] *School of Petrochemical Engineering, Liaoning Petrochemical University, Fushun Liaoning 113001, China.*

[2] *College of Science, Liaoning Petrochemical University, Fushun Liaoning 113001, China.*

* Corresponding authors:

Fengyang Zhao Associate Professor：a406280751@163.com

Yongjian Jiang Lecturer：xiao.jian.happy@163.com

1. kinetic models

Adsorption kinetics is of crucial importance in the study of adsorption mechanisms and rates. The experimental adsorption data were fitted using typical pseudo-first-order kinetics, pseudo-second-order kinetics, and intraparticle diffusion models. The pseudo-first-order kinetics assumes that the diffusion step controls the adsorption process, and the pseudo-second-order kinetics model considers that the chemisorption mechanism controls the adsorption rate. The two model equations are given as follows[S1]:

$$q_t = q_e(1 - e^{-k_1 t}) \qquad \text{(Eq. S1)}$$

$$q_t = k_2 q_e^2 t/(1 + k_2 q_e t) \qquad \text{(Eq. S2)}$$

where q_e (mg g^{-1}) is the calculated equilibrium adsorption capacity, and q_t (mg g^{-1}) represents the amount of MO adsorbed onto MWCNTs@CCh-PEI at time t (min). k_1 (min^{-1}) and k_2 (g mg^{-1} min^{-1}) are the rate constants of the pseudo-first-order and pseudo-second-order kinetic models. The linear graphs are plotted against t with $\ln(q_e-q_t)$ and t/qt, respectively. k_1, k_2, and q_e can be calculated from the intercepts and slopes of the lines.

2. Isotherm models

The Langmuir isotherm model assumes that the adsorption process occurs at specific uniform sites on the adsorbent surface. The adsorbent surface is a uniform single molecular layer after adsorption. The adsorption rate on the adsorbent surface is proportional to the specific surface area of the adsorbent and the concentration of the solution. The following equation can describe the Langmuir isotherm model [S3]:

$$\frac{c_e}{q_e} = \frac{1}{q_m \cdot K_L} + \frac{c_e}{q_m} \qquad \text{(Eq. S3)}$$

where c_e (mg·L^{-1}) and q_e (mg·g^{-1}) are the concentrations of MO and adsorption capacity at equilibrium time. q_m (mg g^{-1}) is identified as the theoretical maximum monolayer adsorption capacity per unit weight of adsorbent. K_L is the Langmuir constant correlated with the adsorption energy and the binding site affinity.

The Freundlich isotherm model assumes that the adsorption process has multiple molecular layers. It applies to non-homogeneous systems on an inhomogeneous

surface. At a constant dye concentration, the amount of dye adsorbed by the adsorbent is not fixed. The Freundlich isotherm model can be expressed by the following equation [S5]:

$$lnq_e = lnK_F + \frac{1}{n} \cdot lnc_e \quad \text{(Eq. S4)}$$

where c_e (mg L^{-1}) and q_e (mg g^{-1}) represent the concentrations of MO and the amount of MO adsorbed onto MWCNTs@CCh-PEI at equilibrium time, and K_F means the Freundlich constant related to the adsorption energy. Note that n is a Freundlich empirical constant related to the adsorption intensity. Based on the Freidlich theory, when $n^{-1} > 1$ and $0 < n^{-1} < 1$, it is defined as difficult and easy to adsorb.

A dimensionless constant R_L is used to represent the critical characteristic of the Langmuir isotherm. The equilibrium parameter, which can be used to predict the feasibility of the adsorbent for dye adsorption ($R_L = 0$ means the adsorption process is irreversible; $0< R_L <1$ represents favorable adsorption; $R_L = 1$ is linear adsorption, and $R_L > 1$ indicates unfavorable adsorption), and the equation can be expressed as [S4]:

$$R_L = \frac{1}{1+K_L \cdot c_0} \quad \text{(Eq. S5)}$$

where K_L is the Langmuir constant, and c_0 (mg·L^{-1}) represents the initial concentration of MO.

3. Thermodynamic models

This experiment investigated the thermodynamic properties of adsorption at different temperatures (303, 313, and 323 K) to further understand the internal energy changes during adsorption. The main thermodynamic parameters, including Gibbs free energy change (ΔG, kJ·mol^{-1}), enthalpy (ΔH, kJ·mol^{-1}), and entropy change (ΔS, kJ·mol^{-1}·K^{-1}), can be calculated from the following equations [S6]:

$$\Delta G = -RTlnK\alpha \quad \text{(Eq. S6)}$$

$$lnK\alpha = \frac{\Delta S}{R} - \frac{\Delta H}{RT} \quad \text{(Eq. S7)}$$

$$K\alpha = \frac{q_e}{c_e} \quad \text{(Eq. S8)}$$

where T (K) is the ambient temperature, and R (8.314 J·mol^{-1} K^{-1}) is the universal

gas constant. q_e ($mg \cdot g^{-1}$) and c_e ($mg \cdot L^{-1}$) are the equilibrium adsorption capacity of MO on the adsorbent and the equilibrium concentration of MO, respectively.

References

[S1].Hong HZ, Sha XF, Cui Y, Sun SY, Huang HY, He ZY, Liu MY, Zhou NG, Zhang XY, Wei Y (2020) Highly efficient removal of iodine ions using MXene-PDA-Ag_2O_x composites synthesized by mussel-inspired chemistry. J Colloid Interface Sci 567: 190-201.

[S2].Theydan SK, Ahmed MJ (2012) Adsorption of methylene blue onto biomass-based activated carbon by $FeCl_3$ activation: Equilibrium, kinetics, and thermodynamic studies. J Anal Appl Pyrolysis 97: 116-122.

[S3].Bayramoglu G, Akbulut A, Liman G, Arica MY (2017) Removal of metal complexed azo dyes from aqueous solution using tris(2-aminoethyl)amine ligand modified magnetic p(GMA-EGDMA) cationic resin: Adsorption, isotherm and kinetic studies. Chem Eng Res Des 124: 85-97.

[S4].Bai LZ, Li ZP, Zhang Y, Wang T, Lu RH, Zhou WF, Gao HX, Zhang SB (2015) Synthesis of water-dispersible graphene-modified magnetic polypyrrole nanocomposite and its ability to efficiently adsorb methylene blue from aqueous solution. Chem Eng J 279: 757-766

[S5].Ma H, Pu SY, Hou YQ, Zhu RX, Zinchenko A, Chu W (2018) A highly efficient magnetic chitosan "fluid" adsorbent with a high capacity and fast adsorption kinetics for dyeing wastewater purification. Chem Eng J 345: 556-565

[S6].Derafa G, Zaghouane-Boudiaf H (2019) Urtica dioica leaves-calcium alginate as a natural, low cost and very effective bioadsorbent beads in elimination of dyes from aqueous medium: Equilibrium isotherms and thermodynamic studies. Int J Biol Macromol 124: 915-921
